# New and Old Indices for Evaluating Heat Stress in an Indoor Environment: Some Considerations. Comment on Kownacki, L.; Gao, C.; Kuklane, K.; Wierzbicka, A. Heat Stress in Indoor Environments of Scandinavian Urban Areas: A Literature Review. *Int. J. Environ. Res. Public Health* 2019, 16 (4), 560. doi:10.3390/ijerph16040560

**DOI:** 10.3390/ijerph16081444

**Published:** 2019-04-23

**Authors:** Francesco Chirico, Nicola Magnavita

**Affiliations:** 1 Health Service Department, State Police, Ministry of Interior, 20162 Milan, Italy; 2 Institute of Public Health., Università Cattolica del Sacro Cuore, 00168 Rome, Italy; nicola.magnavita@unicatt.it; 3 Department of Woman/Child and Public Health, Fondazione Policlinico “A. Gemelli” IRCCS, 00168 Rome, Italy

**Keywords:** indoor environment, heat stress, occupational health, outdoor, public health, thermal risk assessment

## Abstract

In their review, Kownacki et al. showed some practical and easy to use workplace heat indices that are useful for indoor environments, namely the “Wet Bulb Globe Temperature” (WBGT), the “Predicted Heat Strain” (PHS) model, the “Thermal Work Limit” (TWL), the “Equivalent Temperature” (ET) and the thermal comfort index “PMV/PPD”. In this letter, the authors explain why the modified PMV/PPD method together with the indices combining temperature with humidity, such as the “Humidex Index” and the “Heat Index”, could be a more feasible and useful tool for evaluating potential thermal stress in indoor environments for both the occupational and general population.

In their original review, Kownacki et al. [1] explored the connection between the outdoor and the indoor climates in buildings without air conditioning as climate change is likely to increase the risk of heat waves in the future, which can be a potential source of thermal stress for indoor inhabitants. Their review concluded that the literature on this topic is scarce as most of the studies have only focused on the outdoor environment. As shown by the authors, the climate parameters in the outdoor environment, such as temperature, solar radiation and humidity, may be directly related to the indoor conditions of buildings without air conditioning. Therefore, the review showed some practical and easy to use workplace heat indices that are useful for indoor environments, namely the “Wet Bulb Globe Temperature” (WBGT), the “Predicted Heat Strain” (PHS) model, the “Thermal Work Limit” (TWL), the “Equivalent Temperature” (ET) and the thermal comfort index “PMV/PPD”. As stated by Kownacki et al., several studies have found a strong correlation between the outdoor and indoor temperatures although one study [2] found that the relationship between indoor and outdoor temperatures is more complex. On the contrary, many studies focusing on the relationship between the humidity indoors and outdoors produce more consistent results compared to the temperature relationship. For this reason, we should like to mention two further indices that the authors did not include in their review, namely the “Humidex Index” [3], which is used by Canadian meteorologists to describe how hot the weather feels to the average person by combining the effect of heat and humidity and the “Heat Index” [4], which is an easily calculated index that is used by US Government as a measure of how hot it really feels when relative humidity is factored in with the actual air temperature [5]. In indoor environments, the methods suggested by ISO 7730, ISO 7933 and ISO 7243 (1989) are PMV, PHS and WBGT (Wet-Bulb Globe Temperature), respectively. However, these methods have some shortcomings, especially in indoor non-occupational settings. Indeed, it is difficult to calculate PHS for the purpose of protecting the general population. WBGT has limits that depend on the presence or absence of direct sunlight and appreciable ventilation. Finally, PMV can underestimate if there are high humidity rates [6,7,8,9,10,11].

Finally, Kownacki et al. [1] recognized that all indices have limitations as they were developed for healthy working adults. In order to determine the severity of indoor heat, which is relevant to public health, a new heat index needs to be developed or existing thresholds must be adjusted to include vulnerable groups, different uses and daily variations. As Lenzuni et al. have already highlighted [6,12], it is possible to provide extra care for children, elderly, pregnant women, disabled and other ‘weak’ categories, as required by ISO/TS 14415:2005, by setting the highest comfort level of PMV/PPD indexes. In other words, as suggested by Italian guidelines developed by INAIL [6], the A-B-C categories of UNI EN ISO 7730 could be standardized with the four-stage classification suggested by EN 16798-1 and EN 16798-2. In our opinion, this modified PMV/PPD method together with indices combining temperature with humidity could be a more feasible and useful tool for evaluating potentially thermal stress in indoor environments for both the occupational and general population.
